# Supplementary material for: Au-Ag Alloy Nanoshuttle Mediated Surface Plasmon Coupling for Enhanced Fluorescence Imaging
Source: Biosensors (Basel). 2022 Nov 13;12(11):1014. doi: 10.3390/bios12111014 (PMC9688416; doi:10.3390/bios12111014)
Supplement: Supplementary file 1 [file biosensors-12-01014-s001.zip › biosensors-1974294-supplementary.pdf]

biosensors

Supporting Information

Au-Ag Alloy Nanoshuttle Mediated Surface Plasmon Coupling for Enhanced Fluorescence Imaging

Kai-Xin Xie [1,2,†], Zhao Li [2,†], Jia-Hua Fang [2], Shuo-Hui Cao [2,3,*] and Yao-Qun Li [2,*]

[1] College of Chemistry and Materials, Taiyuan Normal University, Jinzhong 030619, China
[2] Department of Chemistry and the MOE Key Laboratory of Spectrochemical Analysis & Instrumentation, College of Chemistry and Chemical Engineering, Xiamen University, Xiamen 361005, China
[3] Department of Electronic Science, Xiamen University, Xiamen 361005, China
* Correspondence: shuohuicao@xmu.edu.cn (S.-H.C.); yaoqunli@xmu.edu.cn (Y.-Q.L.)
† These authors contributed equally to this work.

1. The Synthesized Process of Au-Ag NSs.

The synthesis process mainly refers to a published article by Bai et al. in 2014 (ACS Appl. Mater. Interfaces, 2014, 6, 3331-3340), and the synthesized process is shown below. Original Au NRs were prepared by a seed-mediated method developed by Ye et al. in 2013 (Nano Lett., 2013, 13, 765-771). A stable pH of 8.5 was obtained by mixing 5 mL of purified Au NRs (centrifugation twice at 10,000 rcf for 15 min) in 0.1 M hexadecyltrimethylammonium bromide, 5 mL of 0.2 M glycine acid and 30 μL of 2 M NaOH solutions. Then, 40 μL of $HAuCl_4$ (25 mM) and 0.2 mL of $AgNO_3$ (10 mM) were added under vigorous stirring and incubated at 27 °C. Finally, 0.2 mL of ascorbic acid solution (0.1 M) was added to the solution and stirred for 30 s, and then the whole solution was aged for 1 h. The nanostructures were centrifuged at 7000 rcf for 10 min and dispersed in deionized water.

2. The Extinction Spectrum and XRD Patterns for Au-Ag NSs

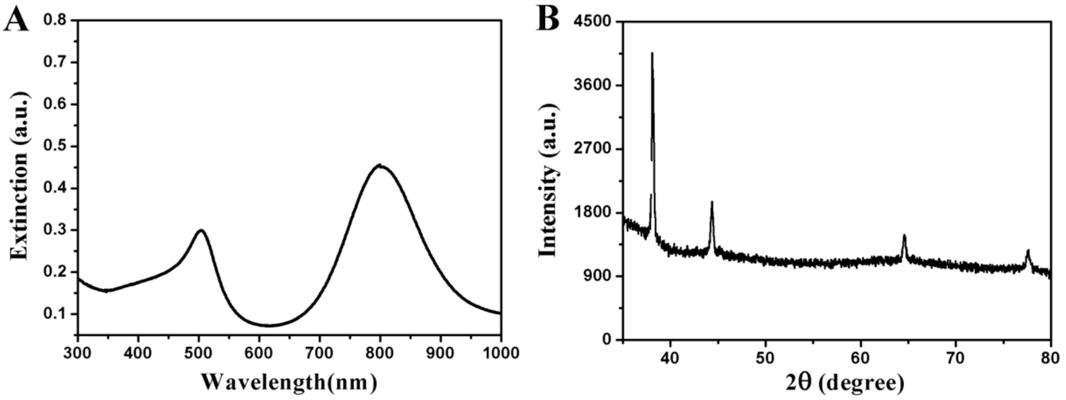

Figure S1. (**A**) The extinction spectrum and (**B**) XRD patterns for Au-Ag NSs.

3. The Excitation Angular Distribution of RhB-PMMA Fluorophore Layer without NSs

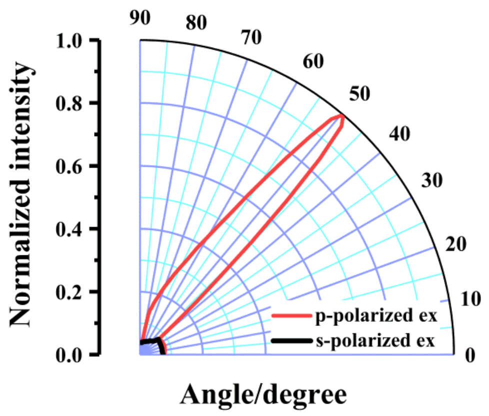

Figure S2. The excitation angular distribution of RhB-PMMA fluorophore layer without NPs from different polarization.

4. The Calculated SPR Curve

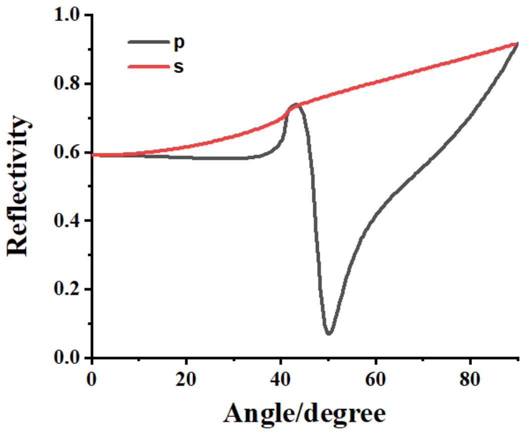

Figure S3. The calculated SPR curve for 561 nm with fluorophore layer about 20 nm.

5. The TEM Image and Mapping Analysis for Au-Ag NSs

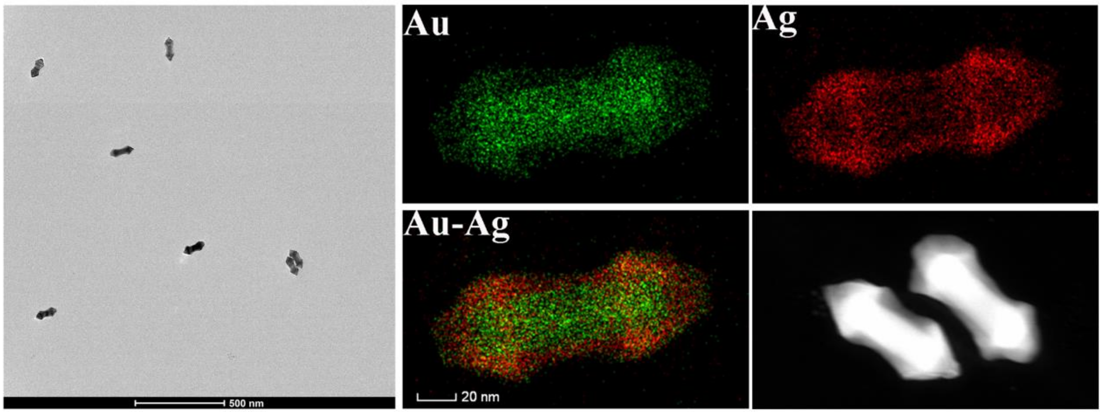

Figure S4. The TEM image and mapping analysis for Au-Ag NSs.

6. The Reproducibility of The NSs Enhanced RhB-PMMA Fluorophore Layer Imaging

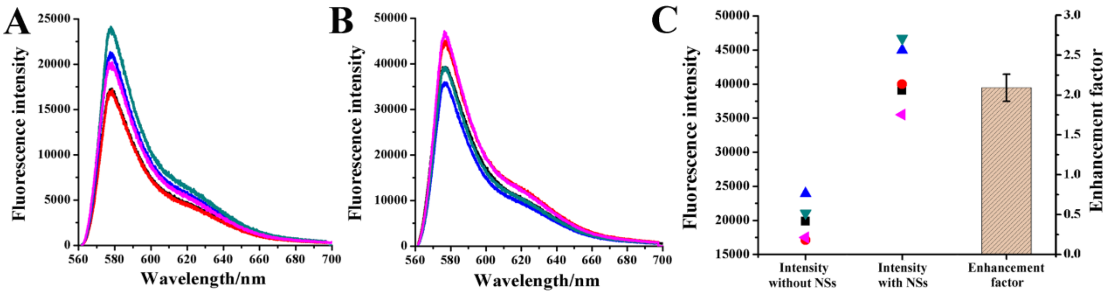

Figure S5. The intensity of RhB-PMMA layer imaging (**A**) without NSs enhancement, (**B**) with NSs enhancement, and (**C**) the reproducibility of enhancement factor.

7. The Normalized Intensity of RhB-PMMA Fluorophore Layer with Different Concentrations of Au-Ag NSs and The Image of RhB-PMMA Fluorophore Layer Modified with High Concentration of Au-Ag NSs

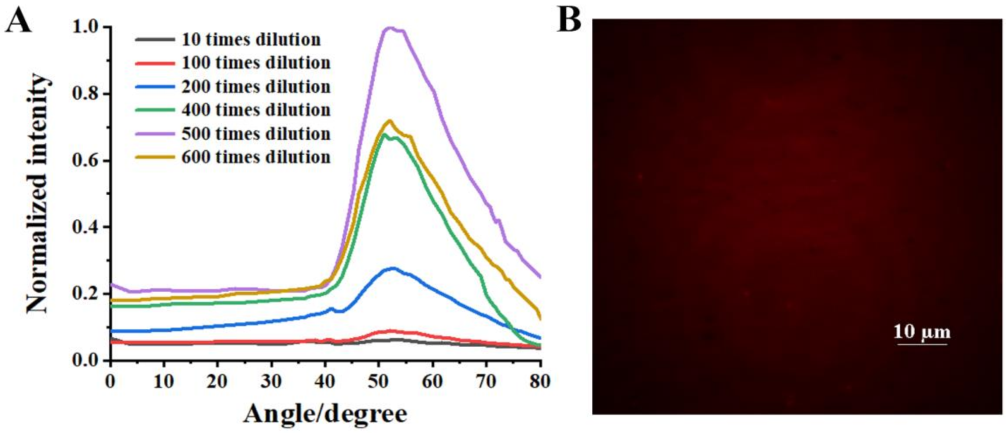

Figure S6. (**A**) The normalized intensity of RhB-PMMA fluorophore layer with different dilution times of Au-Ag NSs and (**B**) the image of RhB-PMMA fluorophore layer with high concentration of Au-Ag NSs (10 times dilution).

8. The excitation angular distribution and images of RhB dyeing cell

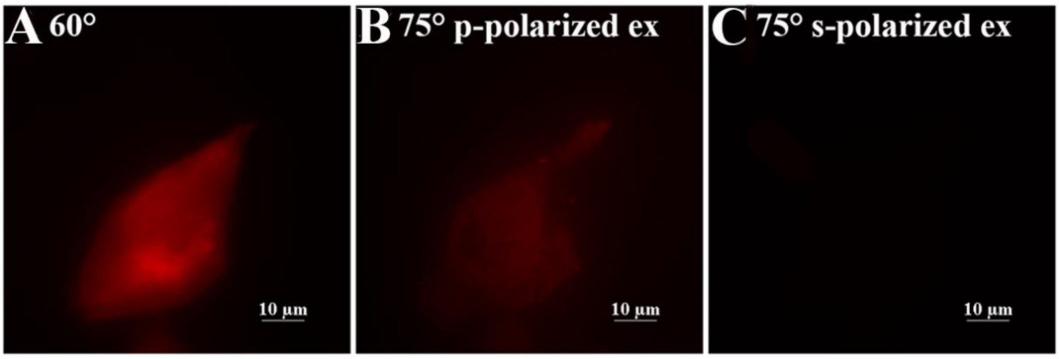

Figure S7. The images from (**A**) critical angle excitation, and the images from SPR excitation with (**B**) p- and (**C**) s-polarization for RhB dyeing cell without NPs.

9. The SBR of The RhB Dyeing Cells Imaging

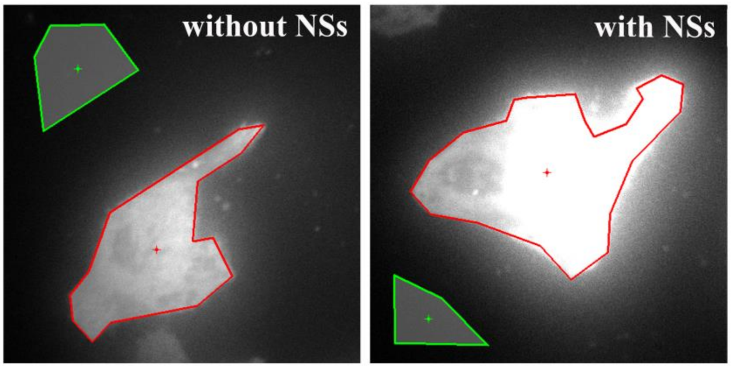

RhB dyeing cell	Without NSs	With NSs
Average signal of cell region	3140	4400
Average signal of background region	450	395
SBR	7.0	11.1

Figure S8. The ROI of RhB dyeing cell imaging without and with NSs, and the related average signal and SBR.

10. The SBR of The DiI Dyeing Cells Imaging

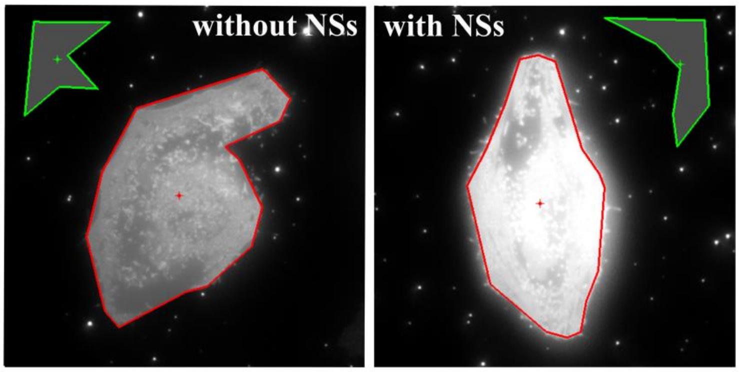

DiI dyeing cell	Without NSs	With NSs
Average signal of cell region	5810	13570
Average signal of background region	258	310
SBR	22.5	43.7

Figure S9. The ROI of DiI dyeing cell imaging without and with NSs, and the related average signal and SBR.

11. The Images Obtained from EPI Mode (Coverslip)

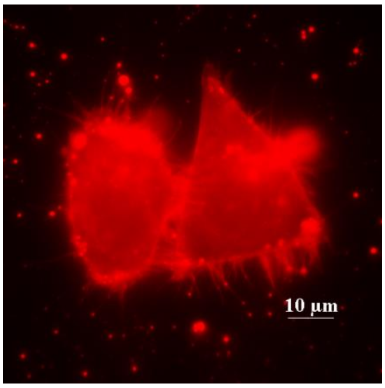

Figure S10. The image obtained from EPI mode on coverslip.
